# An *In Vitro* Synergistic Interaction of Combinations of *Thymus glabrescens* Essential Oil and Its Main Constituents with Chloramphenicol

**DOI:** 10.1155/2014/826219

**Published:** 2014-01-28

**Authors:** Budimir S. Ilić, Branislava D. Kocić, Vojislav M. Ćirić, Olga G. Cvetković, Dragoljub L. Miladinović

**Affiliations:** ^1^Department of Pharmacy, Faculty of Medicine, University of Niš, 18000 Niš, Serbia; ^2^Center for Microbiology, Institute for Public Health, 18000 Niš, Serbia; ^3^Clinic for Endocrinology, Diabetes and Diseases of Metabolism, Clinical Center Niš, 18000 Niš, Serbia; ^4^Center of Chemistry, ICTM, University of Belgrade, 11000 Belgrade, Serbia

## Abstract

The chemical composition and antibacterial activity of *Thymus glabrescens* Willd. (Lamiaceae) essential oil were examined, as well as the association between it and chloramphenicol. The antibacterial activities of geraniol and thymol, the main constituents of *T. glabrescens* oil, individually and in combination with chloramphenicol, were also determined. The interactions of the essential oil, geraniol, and thymol with chloramphenicol toward five selected strains were evaluated using the microdilution checkerboard assay in combination with chemometric methods. Oxygenated monoterpenes were the most abundant compound class in the oil, with geraniol (22.33%) as the major compound. The essential oil exhibited *in vitro* antibacterial activity against all tested bacterial strains, but the activities were lower than those of the standard antibiotic and thymol. A combination of  *T. glabrescens* oil and chloramphenicol produced a strong synergistic interaction (FIC indices in the range 0.21–0.87) and a substantial reduction of the MIC value of chloramphenicol, thus minimizing its adverse side effects. The combinations geraniol-chloramphenicol and thymol-chloramphenicol produced synergistic interaction to a greater extent, compared with essential oil-chloramphenicol association, which may indicate that the activity of the thyme oil could be attributed to the presence of significant concentrations of geraniol and thymol.

## 1. Introduction

Antimicrobial resistance (AMR) represents a rapidly growing public health concern worldwide. AMR has been observed following the introduction of every antimicrobial agent into clinical practice. For example, resistance of the bacterium *Staphylococcus aureus* to penicillin was encountered in hospitals in the mid-1940s, only a few years after the introduction of penicillin [1]. A multifaceted approach is needed to combat AMR, including the discovery of novel antimicrobial drugs and/or new methodological concepts.

Many studies have shown significant antibacterial activity of essential oils against a wide range of resistant microbial strains [2]. The antibacterial activity of essential oils could reflect all the molecules present or only those present in high amounts. For the same reasons, no particular bacterial resistance or adaptation to essential oils has been described and secondary effects have not been confirmed. To enhance the efficacy of antimicrobial drugs and avoid their potentially toxic side effects, their combination with an essential oil may be an innovative alternative and promising strategy [3].

The genus *Thymus* contains about 350 species, most commonly used in traditional medicine as antibacterial and antifungal remedies [4]. The Serbian flora recognizes 30 species of the *Thymus* genus, with more than 60 varieties [5].

Given the importance of *Thymus* species as useful antibacterial remedies, the aim of the present study was to examine the chemical composition and antibacterial effect of the essential oil of* Thymus glabrescens* (thyme), as well as the association between it and chloramphenicol. The antibacterial activities of geraniol and thymol, the main active principles of thyme oil, in combination with chloramphenicol were also determined.

## 2. Materials and Methods

### 2.1. Plant Material and Chemicals

The aerial parts of *Thymus glabrescens* Willd. (Lamiaceae) were collected in June 2011 from natural populations at the Kravlje village, southeast Serbia. A voucher specimen, with the accession number 16642, is deposited at the Herbarium of the Department of Botany, Faculty of Biology, University of Belgrade Herbarium Code BEOU. All chemicals, reagents, and standards were of analytical reagent grade and were purchased from the Sigma-Aldrich Chemical Company.

### 2.2. Oil Isolation

The aerial parts of the plant (dried and ground) were subjected to hydrodistillation for 4 h, using a Clevenger-type apparatus to obtain the oil. The resulting essential oil was dried over anhydrous sodium sulphate and stored at 4°C.

### 2.3. Chemical Analysis

Quantitative and qualitative data of the essential oil were obtained by gas chromatography (GC) and gas chromatography/mass spectrometry (GC-MS) analyses.

### 2.4. Gas Chromatography

The GC analysis of the oil was performed on a GC HP-5890 II apparatus, equipped with the split-splitless injector, an HP-5MS capillary column (30 m × 0.25 mm, 0.25 *μ*m film thickness) using helium as the carrier gas (1 mL/min), and an FID. Operating conditions were as follows: injector temperature 250°C, interface temperature of 280°C, temperature program from 50°C (3 min) to 250°C at a rate of 3°C/min.

### 2.5. Gas Chromatography/Mass Spectrometry

GC-MS analyses were performed on an Agilent Technologies apparatus, Model GS 6890N at 70 eV coupled with a mass selective detector MSD 5975C, under the same gas-chromatographic conditions.

### 2.6. Identification of Compounds

Identification of the compounds was based on comparison of arithmetic retention indices (applying calibrated automated mass spectral deconvolution and identification system software AMDIS ver. 2.64) in combination with the selective ion analysis (SIA) resolution method by Tan et al. [6], comparison with the spectral data from the available literature [7], and comparison of their mass spectra to those from Wiley 275 and NIST/NBS libraries using various search engines. The retention indices were obtained by coinjection with a standard aliphatic hydrocarbons C_7_–C_40_ mixture.

### 2.7. Antibacterial Testing

The activity of the essential oil samples was tested towards 13 different bacteria. Gram-negative bacteria were represented by *Escherichia coli* ATCC 25922, *Salmonella enteritidis* ATCC 13076, *Klebsiella pneumoniae* ATCC 10031, *Klebsiella pneumoniae* ATCC 700603, *Proteus mirabilis* ATCC 12453, *Pseudomonas aeruginosa* ATCC 9027, *Pseudomonas aeruginosa* ATCC 27853, and *Enterobacter aerogenes* ATCC 13048, while the researched Gram-positive strains were* Enterococcus faecalis* ATCC 19433, *Bacillus cereus* ATCC 11778, *Staphylococcus aureus* ATCC 25923, *Staphylococcus aureus* ATCC 29213, and *Listeria monocytogenes* ATCC 15313.

The inocula of the bacterial strains were prepared from overnight broth cultures and the suspensions were adjusted to 0.5 McFarland standard turbidity (corresponding to 10^8^ CFU/mL, depending on genera-consensus standard by the Clinical and Laboratory Standards Institute) [8].

### 2.8. Microwell Dilution Assay

A broth microdilution method was used to determine the minimum inhibitory concentration (MIC) and minimum bactericidal concentration (MBC) according to the Clinical and Laboratory Standards Institute [8]. Serial double dilutions of the tested oil, as well as the geraniol and thymol, were prepared in 70.0% ethanol and then transferred into a 96-well microtiter plate over the concentration range of 0.025–50.0 *μ*L/mL in inoculated nutrient broth. The final volume was 100 *μ*L and the final bacterial concentration was 10^7^ CFU/mL in each well. The plate was incubated for 24 h at 37°C. All experiments were performed in triplicate. Two controls were included, a medium with solvent/ethanol (negative control) and a medium with antibiotic chloramphenicol (positive control). Bacterial growth was determined by adding 20 *μ*L of an aqueous 0.5% triphenyl tetrazolium chloride (TTC) solution. The minimal inhibitory concentration was defined as the lowest concentration of the oil inhibiting visible growth (red collared pellet on the bottom of the wells after the addition of TTC), while the minimal bactericidal concentration was defined as the lowest oil concentration killing 99.9% of the bacterial cells. To determine the MBC, the broth was taken from each well without visible growth and inoculated in Mueller Hinton agar (MHA) for 24 h at 37°C.

### 2.9. Microdilution Checkerboard Assay

The microdilution checkerboard method is the technique used most frequently to assess antimicrobial combinations *in vitro* [9, 10]. Dilutions of *T. glabrescens* oil, geraniol, thymol, and the examined antibiotic were made for evaluation of their combined interactions. The type of interaction was studied on the *E. coli* ATCC 25922, *K. pneumonia* ATCC 700603, *P. mirabilis* ATCC 12453, *P. aeruginosa* ATCC 27853, and *S. aureus* ATCC 29213. These strains were selected based on their importance in frequently occurring infections. Dilutions from the logarithmic-growth phase of the bacterial culture were prepared and distributed into microtiter trays containing combinations of varying concentrations: chloramphenicol-*T. glabrescens* oil, chloramphenicol-geraniol, and chloramphenicol-thymol. The CLSI [8] guidelines were used to ensure that accurate microbiological assay and transfer techniques were followed. The inoculated trays were incubated at 37°C for 24 h and then evaluated for bacterial growth. Determinations of essential oil-antibiotic interactions were based on the median-effect principle and multiple drug effect equation as described by Chou and Talalay [11]. Three effects can be highlighted: synergetic, additive, or antagonist as a result of the combined effects of the *T. glabrescens* oil, geraniol, thymol, and chloramphenicol. For quantitative purposes the concept of fractional inhibitory concentrations (FIC) is frequently used. In order to assess the activities of combinations of two drugs that are mutually nonexclusive (have different modes of action), the FIC indices were calculated as

(1) FIC=MICA combinationMICA alone+MICB combinationMICB alone+MICA combination×MICB combinationMICA alone×MICB aloneFIC=FICA+FICB+FICA×FICB,

where MIC_A_ are the minimum concentrations of the essential oil, geraniol, and thymol, while MIC_B_ are the minimum concentrations of the examined antibiotic that inhibited the bacterial growth, respectively. The FIC indices were calculated using CalcuSyn (Biosoft), and the results were interpreted as follows: synergistic (<0.90), additive (0.90 ≤ FIC ≤ 1.10), or antagonistic (>1.10) [12].

### 2.10. Statistical Analysis of Data

The experimental data (FIC values) were analyzed by chemometric methods: principal components analysis (PCA) and hierarchical cluster analysis (HCA), using Mathworks MATLAB.

## 3. Results

To eliminate any kind of subjective analysis, interpretations and discussions of the results, presented by tables and/or graphics, and the chemometric methods: principal component analysis, and hierarchical cluster analysis were employed. Furthermore, the use of chemometric methods allows the maximum number of experimental results to be obtained and moreover enables the detection of connections, similarities, and differences among variables in the researched experimental system [4].

### 3.1. Chemical Composition of the Essential Oil

The yield of *T. glabrescens* essential oil was 0.59% (w/w). Based on GC and GC-MS analysis of the thyme essential oil, 56 components were identified that represented 97.76% of the total detected constituents (Table 1). The components of *T. glabrescens* essential oil were separated into six classes, that is, monoterpene hydrocarbons, oxygenated monoterpenes, sesquiterpene hydrocarbons, oxygenated sesquiterpenes, phenolic compounds, and others. The oxygenated monoterpenes were the most abundant compound class in the oil (57.14%), and they were dominated by geraniol (22.33%), geranyl acetate (19.38%), and linalool (5.49%). The group of phenolic compounds (14%) was mainly dominated by thymol (13.79%).

### 3.2. Antibacterial Activity

The essential oils were tested for their antibacterial activity by broth microdilution method to determine the MIC and MBC values against thirteen model bacteria (Table 2). The results from the antibacterial assay show that thyme essential oil possessed antimicrobial activities against all the tested microorganisms with MIC values ranging from 627.1 to 10033.6 *μ*g/mL and MBC values from 627.1 to 20067.2 *μ*g/mL. Gram-positive bacteria were generally found to be more sensitive than the Gram-negative ones. Geraniol was active with MIC values ranging from 1386.8 to 5547.2 *μ*g/mL and MBC values from 1386.8 to 11094.4 *μ*g/mL. Thymol exhibited antibacterial activity with MIC values ranging from 24.4 to 3123.2 *μ*g/mL and MBC values from 24.4 to 6246.4 *μ*g/mL. The reference antibiotic was active in the range of concentration 1 to 2048 *μ*g/mL.

### 3.3. Interactions between the Essential Oil, Geraniol, and Thymol with the Reference Antibiotic

**Figure 1:**
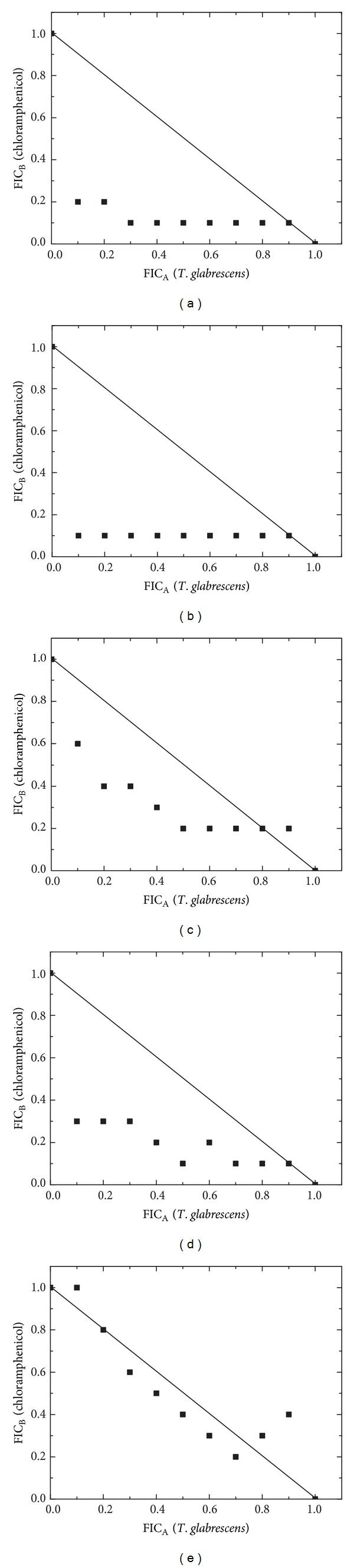
The derived isobolograms for the interaction of *T. glabrescens* oil-chloramphenicol and their treatment outcomes against the following: (a) *E. coli* ATCC 25922, (b) *K. pneumoniae* ATCC 700603, (c) *P. mirabilis* ATCC 12453, (d) *P. aeruginosa* ATCC 27853, and (e) *S. aureus* ATCC 29213.

**Figure 2:**
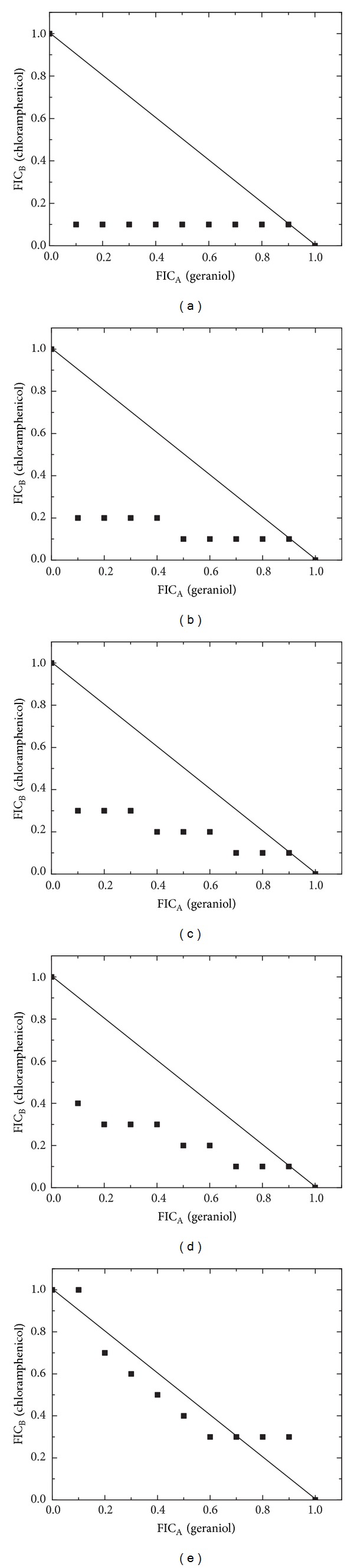
The derived isobolograms for the interaction of geraniol-chloramphenicol and their treatment outcomes against the following: (a) *E. coli* ATCC 25922, (b) *K. pneumoniae* ATCC 700603, (c) *P. mirabilis* ATCC 12453, (d) *P. aeruginosa* ATCC 27853, and (e) *S. aureus* ATCC 29213.

**Figure 3:**
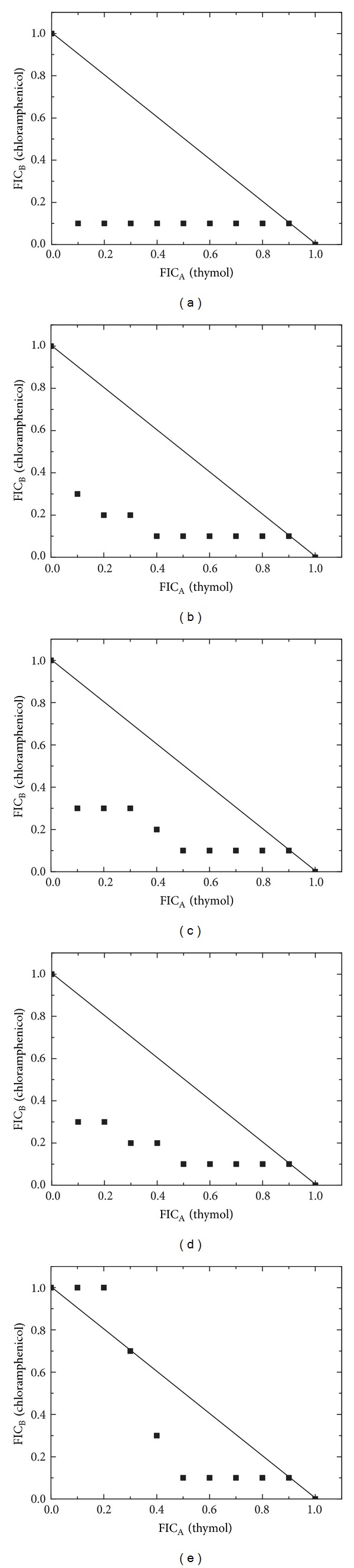
The derived isobolograms for the interaction of thymol-chloramphenicol and their treatment outcomes against the following: (a) *E. coli* ATCC 25922, (b) *K. pneumoniae* ATCC 700603, (c) *P. mirabilis* ATCC 12453, (d) *P. aeruginosa* ATCC 27853, and (e) *S. aureus* ATCC 29213.

The results of the possible interactions between the essential oil, geraniol, and thymol with the reference antibiotic are given in Figures 1–3.

Of the 45 combinations of* T. glabrescens* essential oil-chloramphenicol, 25 (55.6%) showed synergism, while 14 (31.1%) had an additive and 6 (13.3%) had an antagonistic effect (Figure 1). Studies on *E. coli* ATCC 25922 and *K. pneumoniae* ATCC 700603 showed a synergistic pattern for seven ratios (FIC indices in the range 0.21–0.87). Synergy was also noted when tested against *P. aeruginosa* ATCC 27853 (six ratios, FIC indices in the range 0.43–0.87) and *P. mirabilis* ATCC 12453 (five ratios, FIC indices in the range 0.68–0.82). Combinations with *S. aureus* ATCC 29213 indicated additive (five ratios) and antagonistic (four ratios) effects.

From all the tested combinations of geraniol-reference antibiotic (Figure 2), 26 (57.8%) showed synergism, 15 (33.3%) had an additive effect, and 4 (8.9%) had an antagonistic effect. Studies on *E. coli* ATCC 25922 and *K. pneumoniae* ATCC 700603 showed a synergistic pattern for seven ratios (FIC indices in the range 0.21–0.87). Synergy was also noted when tested against *P. mirabilis* ATCC 12453 and *P. aeruginosa* ATCC 27853 (six ratios, FIC indices in the range 0.43–0.87). Combinations with *S. aureus* ATCC 29213 indicated additive (five ratios) and antagonistic (four ratios) effects.

The combination profiles of thymol with chloramphenicol are presented in Figure 3. A predominantly synergistic profile was noted against all the studied pathogens. Synergy was best noted for 32 (71.1%) ratios, an additive effect was recorded for 10 (22.2%), ratios and three combinations (6.7%), against *S. aureus* ATCC 29213, exhibited an antagonistic effect. To evaluate the correlation among the antibacterial activities of the essential oil-chloramphenicol, geraniol-chloramphenicol, and thymol-chloramphenicol combinations, the FIC values were subjected to PCA and HCA analysis.

### 3.4. PCA and HCA Analysis of the Total FIC Indices of the Essential Oil, Geraniol, Thymol, and Chloramphenicol Combinations

**Figure 4:**
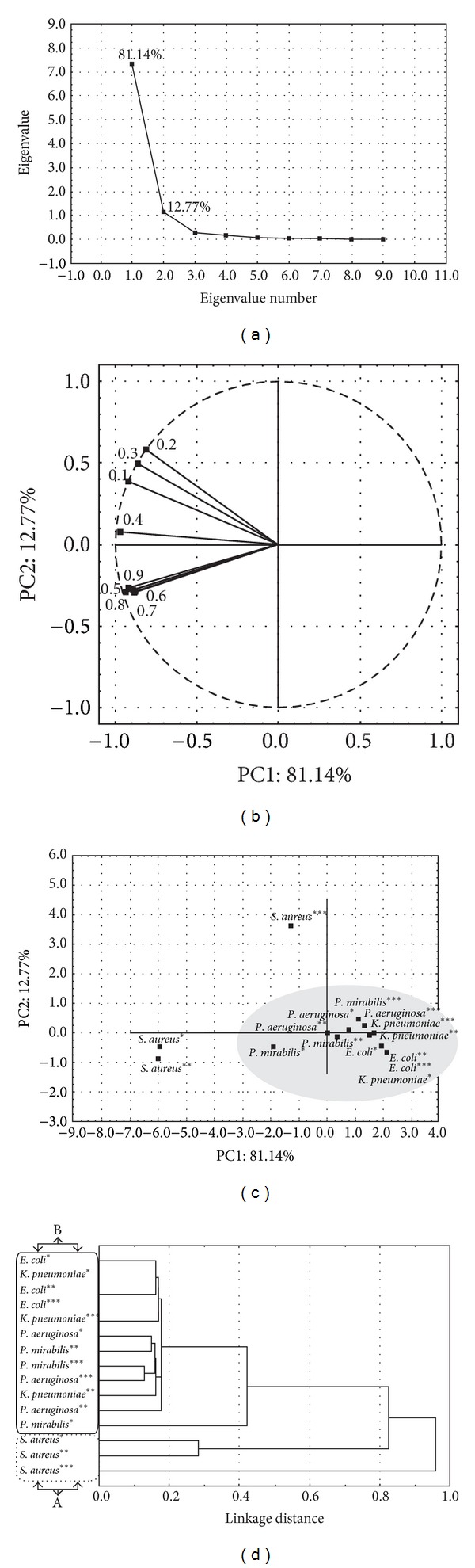
PCA and HCA of the antibacterial activity of the studied combinations {*T. glabrescens* oil-chloramphenicol (*); geraniol-chloramphenicol (**); thymol-chloramphenicol (***)} based on their FIC values: (a) eigenvalues of the correlation matrix, (b) the loading plot of the responsible FIC values, (c) the score plot of the examined bacteria, and (d) the corresponding dendrogram. The examined FIC values are presented in Figures 1–3.

PCA and HCA were applied on all FIC data (Figures 1–3) in order to evaluate similar antibacterial behaviour among studied combinations. According to the eigenvalues of the obtained correlation matrix, the PC1 horizontal axis explained 81.14% of the total variance among the tested interactions, while the PC2 vertical axis showed a further 12.77% (Figure 4(a)). The loading plot (Figure 4(b)) illustrates the influence of the FIC values, marked by FIC_A_ equivalents, responsible for the classification of the interaction in the score plot (Figure 4(c)). Based on the Euclidean distance and dissimilarity ≥0.42 (Figure 4(d)), the HCA method indicated two groups of interaction (A and B). Group A, constituted only by *S. aureus* ATCC 29213, was characterized mainly by strong antagonistic interactions with the applied combinations. In this group, only the association thymol-chloramphenicol showed some percent of synergistic interaction. In contrast, in group B, formed by the rest of the examined bacteria strains and studied combinations, mainly synergistic or additive interactions were detected.

## 4. Discussion

The essential oil of *T. glabrescens* from southeast Serbia belongs to the geraniol/geranyl acetate/thymol chemotype [13]. Chemical polymorphism of the essential oils is a characteristic of the species of the *Thymus* genus. Except for genetic factors, environmental conditions also have an influence on the chemical composition of an essential oil. It was established that the production of phenolic compounds is favoured in warmer and drier climatic zones, while the other, nonphenolic compounds usually accumulate in higher quantities in cooler and damper areas [14]. Geraniol is the dominant component of *T. glabrescens* essential oil from Romania [15]. In Hungarian *T. glabrescens* essential oil, the major compounds were sesquiterpenes: germacrene D, *β*-caryophyllene, and caryophyllene oxide [13].

The release of cellular content in the treated bacteria led to the hypothesis that the first effect of an essential oil is membrane disruption. However, the fact that some interaction with other targets of the bacterial cell might play a key role in the observed antibacterial effects of the essential oil should not be ignored [16]. The antibacterial activity of *T. glabrescens* essential oil displayed variation among the different bacteria species but remained lower than the activities of the standard antibiotic and thymol. A correlation of the antibacterial activity of the oil and its chemical composition suggests that the activity of the oil could be attributed to the presence of significant concentrations of geraniol and thymol. Therefore, it was decided to study also the antibacterial activity of thymol and geraniol individually and in combination with chloramphenicol.

The essential oil of* T. glabrescens* from Romania inhibited microbial growth in a range of concentrations from 10.8 to 27 *μ*L/mL [15]. As noted, the main antibacterial agent of the *T. glabrescens* essential oil from southeast Serbia is not only geraniol but also thymol (13.79%); together they represent 36.12% of* T. glabrescens* essential oil. It is interesting to emphasize that the antibacterial activity of *T. pulegioides* essential oil with geraniol (66.59%) as the major constituent is significantly higher in comparison with antibacterial activity of *T. glabrescens* essential oil towards the same bacterial strains [17].

In a study of the inhibitory activity of terpenes on slime producing methicillin resistant strains, the authors found MIC values for geraniol of 5.8 mg/mL against the methicillin resistant *S. aureus* (MRSA) and 23.4 mg/mL against methicillin sensitive* S. aureus* (MSSA) [18]. In the same study, thymol exhibited inhibitory activities against MRSA and MSSA strains with an MIC value of 3.17 mg/mL. These values are generally higher compared to the values of the antibacterial activity of geraniol and thymol found in the present research.

In the current investigation, it was confirmed that Gram-positive bacteria were more sensitive with all tested antibacterial agents than Gram-negative ones. Most Gram-negative bacteria are intrinsically less susceptible to many antibiotics than are Gram-positive bacteria. This difference could be explained by the presence of an outer membrane in Gram-negative bacteria. The structure and composition of the layer of cells differ greatly between bacteria. On the outer envelope, the cells may have polysaccharide capsules or protein layers which protect bacteria under unfavourable conditions and affect their adhesion [19].

The interaction of essential oils with antibiotics is one of the novel ways to overcome bacterial resistance. Essential oils are combined with antibiotics in order to improve the antimicrobial effect and to reduce the required antibiotic concentration [20]. In the present study, the antimicrobial activity of *T. glabrescens* essential oil was evaluated in association with chloramphenicol on five bacterial strains. The combination of thyme oil and chloramphenicol against all the tested bacteria, except *S. aureus* ATCC 29213, exhibited a predominantly synergistic effect and decreased the MIC value of chloramphenicol 10-fold (5-fold for *P. mirabilis* ATCC 12453). Based on the present analyses, it can be assumed that in research of the antibacterial effects of essential oil-antibiotic combinations, the choice of Gram-negative or Gram-positive bacterial species is not decisively significant. In other words, the proper essential oil-antibiotic association will act equally stronger or weaker against all Gram-positive and Gram-negative bacterial strains. In this case, the outer membrane of the Gram-negative bacteria is not a predominant factor of their resistance.

The essential oil of *P. graveolens* and its main components (geraniol and citronellol) exhibited strong synergism with norfloxacin against* B. cereus* and *S. aureus* with FIC indices of 0.50, 0.37, and 0.38, respectively [21]. According to Prashara et al. [22], the antimicrobial action of *Cymbopogon martinii* essential oil (mainly attributed to its geraniol content) against *S. cerevisiae* occurs via a two-step process. The first step involves the passive entry of the oil into the plasma membrane in order to initiate membrane disruption. The second step is reaction with the active sites of the enzymes or action as an H^+^ carrier, thereby depleting the adenosine triphosphate pool.

There are some generally accepted mechanisms of antibacterial interaction that produce synergism, including inhibition of protective enzymes, combination of membrane active agents, sequential inhibition of common biochemical pathways, and the use of membranotropic agents to enhance the diffusion of other antimicrobials [23]. The results obtained in the present study indicate that chloramphenicol, not currently used as a therapeutic agent against Gram-negative bacteria, in combination with an appropriate essential oil, has significant antimicrobial activity, especially against Gram-negative bacteria. Moreover, its minimum effective dose is significantly reduced, and consequently possible toxic side effects are decreased.

The results for the antibacterial activity of a combination of geraniol-chloramphenicol are very similar to the results for a combination of thyme oil-chloramphenicol. The difference is in the increased percentage of interactions that produce synergistic and additive effects, with a decrease in the percentage of antagonistic effects. The combination of geraniol and chloramphenicol against all the tested bacteria, except *S. aureus* ATCC 29213, exhibited predominantly synergistic effects and decreased the MIC value of chloramphenicol 10-fold. The associations of geraniol with penicillin against MRSA and *E. coli *were shown to be indifferent, independently of each antimicrobial activity when they were used alone [24]. In a study of changes in the antibacterial activity and the mode of action of farnesol against *S. aureus *when geraniol was added to a bacterial suspension, the authors assumed that geraniol increased the growth-inhibitory activity of farnesol but suppressed its ability to damage cell membranes, which is one of the predominant features of the growth-inhibitory activity of farnesol. Their results revealed that terpenes might interact with each other and with bacterial cells to increase or decrease the antibacterial activity of each other [25]. Geraniol significantly increased the efficacy of chloramphenicol by targeting efflux mechanisms and produced significant restoration of susceptibility of the multidrug resistance strain EAEP289 to chloramphenicol by as much as 16-fold [26]. Combinations of geraniol and norfloxacin toward *B. cereus* and *S. aureus* exhibit synergistic effects with FIC indices of 0.50 [21]. The present findings and published data led to the speculation that the antibacterial effect of geraniol may result through interaction with the membrane structure and the function of the bacteria. Furthermore, geraniol might cross the cell membranes penetrating into the interior of the cell and interacting with intracellular sites critical for antibacterial activity [27].

The combination of thymol and chloramphenicol against all the tested bacteria exhibited a predominantly synergistic effect and decreased the MIC value of chloramphenicol 10-fold. Antagonism (only three combinations) was evidenced only against *S. aureus* ATCC 29213. Studies on the antibacterial action of thymol showed that it can cause a disturbance of the cytoplasmic membrane, disrupting the proton motive force, electron flow, and coagulation of cell contents. Thymol also impaired the citrate metabolic pathway and affected the enzymes involved in the synthesis of ATP [28]. The results obtained with the combinations of thymol-penicillin toward MRSA were antagonistic, while association between thymol and penicillin against* E. coli *showed synergistic activity with FIC values of 0.15 [24]. It could be argued that these results correspond to the results of the present research. If the results obtained from the study of the antibacterial activity of geraniol-chloramphenicol and thymol-chloramphenicol associations are compared, a similar pattern can be found. This leads to the speculation that geraniol and thymol in combination could not show any antagonistic effect.

If all combinations of the examined essential oil, geraniol, and thymol with chloramphenicol towards the five bacterial strains are taken into consideration, a possible hypothesis is that the components of thyme essential oil, with the geraniol and thymol as the main active principles, favour the mechanism of action of chloramphenicol, the main effect of which is inhibition of the bacterial enzyme peptidyl transferase, thereby preventing the growth of the polypeptide chain during protein synthesis [29]. It could be stated that all associations against *S. aureus* ATCC 29213 were characterized by a number of ratios of antagonistic interactions. In the PCA and HCA analyses this strain stands out and forms a separate group. In contrast, the other combinations exhibited mostly synergistic or additive interactions toward the other bacterial strains, which may indicate, an already supposed assumption, that the activity of the thyme oil could be attributed to the presence of significant concentrations of geraniol and thymol.

## 5. Conclusions

In the present study, the chemical composition of *T. glabrescens* essential oil was examined and a correlation among the antibacterial activities of the essential oil-chloramphenicol, geraniol-chloramphenicol, and thymol-chloramphenicol combinations was realized by the utilization of chemometric methods. It was shown that oxygenated monoterpenes, with geraniol as the dominant constituent, were the most abundant compound class of the essential oil of *T. glabrescens* from Southeast Serbia. The researched essential oil exhibited *in vitro* antibacterial activity against all the tested bacterial strains, but the activities were lower than those of the standard antibiotic and thymol. The combination of thyme oil and chloramphenicol produced predominantly synergistic interactions and substantial reductions in the MIC values of chloramphenicol against Gram-negative bacteria, the pharmacological treatment of which is very difficult nowadays. The combinations geraniol-chloramphenicol and thymol-chloramphenicol produced synergistic interaction to a greater extent, compared with the essential oil-chloramphenicol association. All the examined combinations reduced the minimum effective dose of the antibiotic and, consequently, minimized its adverse side effects.

## Figures and Tables

**Table 1:** Composition of the essential oil of *T. glabrescens*.

Component	RT^a^ (min)	AIL^b^	AIE^c^	*T. glabrescens* (%)
Monoterpene hydrocarbons				**11.07**
*α*-Thujene	8.161	924.0	925.0	0.33
*α*-Pinene	8.400	932.0	932.1	0.29
Camphene	8.951	946.0	948.4	0.15
Sabinene	9.734	969.0	971.6	0.09
*β*-Pinene	9.897	974.0	976.5	0.10
Myrcene	10.320	988.0	989.0	0.59
*α*-Phellandrene	10.909	1002.0	1006.1	0.10
3-Carene	10.984	1008.0	1007.9	0.03
*α*-Terpinene	11.294	1014.0	1016.6	0.60
o-Cymene	11.620	1022.0	1025.6	4.73
Limonene	11.751	1024.0	1029.2	0.81
*β*-*cis*-Ocimene	11.980	1032.0	1035.6	0.15
*β*-*trans*-Ocimene	12.362	1044.0	1046.1	0.18
*γ*-Terpinene	12.822	1054.0	1058.7	2.75
Terpinolene	13.767	1086.0	1084.8	0.17
Oxygenated monoterpenes				**57.14**
Eucalyptol	11.861	1026.0	1032.3	0.56
*trans*-Linalool oxide	13.828	1084.0	1086.5	0.03
Linalool	14.437	1095.0	1103.2	5.49
*α*-Thujone	14.603	1101.0	1107.8	0.38
*cis*-p-Mentha-2,8-dienol	15.697	1133.0	1138.4	0.01
Borneol	16.956	1165.0	1173.0	0.47
4-Terpineol	17.249	1174.0	1181.1	0.47
*α*-Terpineol	17.790	1186.0	1196.1	0.79
*trans*-Dihydrocarvone	18.088	1200.0	1204.5	0.02
Nerol	18.842	1227.0	1226.3	1.18
Isobornyl formate	18.990	1235.0	1230.5	2.87
Neral	19.311	1235.0	1239.8	2.25
Geraniol	19.936	1249.0	1257.8	22.33
Geranial	20.396	1264.0	1271.0	0.50
Bornyl acetate	20.924	1287.0	1286.3	0.20
Nerol acetate	23.364	1359.0	1359.2	0.21
Geranyl acetate	24.244	1379.0	1385.8	19.38
Sesquiterpene hydrocarbons				**14.56**
*α*-Cubebene	23.065	1345.0	1350.2	5.51
*α*-Copaene	23.927	1374.0	1376.2	0.03
*β*-Elemene	24.409	1389.0	1390.7	0.09
*β*-Caryophyllene	25.384	1417.0	1421.3	1.04
*α*-*trans*-Bergamotene	25.770	1432.0	1433.6	0.05
Aromadendrene	25.952	1439.0	1439.4	0.12
(Z)-*β*-Farnesene	26.376	1440.0	1452.9	0.22
*α*-Humulene	26.487	1452.0	1456.4	0.13
*γ*-Muurolene	27.083	1478.0	1475.4	0.16
Germacrene D	27.302	1484.0	1482.4	1.57
*β*-Selinene	27.567	1489.0	1490.8	0.14
Bicyclogermacrene	27.734	1500.0	1496.2	1.01
*β*-Bisabolene	28.153	1505.0	1510.0	4.08
*γ*-Cadinene	28.258	1513.0	1513.6	0.11
*δ*-Cadinene	28.410	1522.0	1518.7	0.21
*β*-Sesquiphellandrene	28.563	1521.0	1523.9	0.09
Oxygenated sesquiterpenes				**0.37**
Spathulenol	30.140	1577.0	1577.0	0.29
Caryophyllene oxide	30.300	1582.0	1582.3	0.08
Phenolic compounds				**14.00**
Thymol	21.350	1289.0	1298.6	13.79
Carvacrol	21.499	1298.0	1303.0	0.19
Eugenol	23.155	1356.0	1352.9	0.02
Others				**0.62**
*trans*-2-Hexenal	5.987	846.0	850.2	0.03
1-Octen-3-ol	10.010	974.0	979.8	0.45
3-Octanol	10.596	988.0	997.2	0.14

Total				**97.76**

^a^RT: retention time; ^b^AIL: arithmetic (retention) index-literature data, and ^c^AIE: arithmetic (retention) index experimentally determined on HP-5MS column.

**Table 2:** Antibacterial activity of *T. glabrescens* essential oil, chloramphenicol, geraniol, and thymol (*μ*g/mL).

Number	Bacterial species	*T. glabrescens *		Chloramphenicol		Geraniol		Thymol	
		MIC	MBC	MIC	MBC	MIC	MBC	MIC	MBC
1	*Escherichia coli* ATCC 25922	2508.4	5016.8	128.0	512.0	1386.8	2773.6	1561.6	1561.6
2	*Salmonella enteritidis* ATCC 13076	627.1	627.1	4.0	8.0	2773.6	2773.6	24.4	24.4
3	*Klebsiella pneumoniae* ATCC 10031	1254.2	1254.2	2.0	2.0	2773.6	5547.2	390.4	390.4
4	*Klebsiella pneumoniae* ATCC 700603	5016.8	10033.6	512.0	1024.0	2773.6	5547.2	1561.6	3123.2
5	*Proteus mirabilis* ATCC 12453	1254.2	2508.4	4.0	64.0	1386.8	1386.8	1561.6	1561.6
6	*Pseudomonas aeruginosa* ATCC 9027	10033.6	20067.2	4.0	16.0	5547.2	11094.4	1561.6	1561.6
7	*Pseudomonas aeruginosa* ATCC 27853	5016.8	5016.8	1024.0	2048.0	2773.6	2773.6	3123.2	6246.4
8	*Enterobacter aerogenes* ATCC 13048	5016.8	5016.8	1.0	1.0	5547.2	5547.2	195.2	195.2
9	*Enterococcus faecalis* ATCC 19433	2508.4	2508.4	2.0	4.0	1386.8	1386.8	195.2	195.2
10	*Bacillus cereus* ATCC 11778	627.1	1254.2	1.0	4.0	1386.8	1386.8	24.4	24.4
11	*Staphylococcus aureus* ATCC 25923	627.1	627.1	1.0	8.0	2773.6	2773.6	97.6	97.6
12	*Staphylococcus aureus* ATCC 29213	2508.4	2508.4	8.0	32.0	2773.6	2773.6	780.8	1561.6
13	*Listeria monocytogenes* ATCC 15313	627.1	1254.2	8.0	8.0	2773.6	2773.6	97.6	97.6
